# A Dynamically Consistent ENsemble of Temperature at the Earth surface since 1850 from the DCENT dataset

**DOI:** 10.1038/s41597-024-03742-x

**Published:** 2024-08-30

**Authors:** Duo Chan, Geoffrey Gebbie, Peter Huybers, Elizabeth C. Kent

**Affiliations:** 1 School of Ocean and Earth Science, University of Southampton, Southampton, UK; 2 Department of Physical Oceanography, Woods Hole Oceanographic Institution, Woods Hole, 02543 USA; 3 Department of Earth and Planetary Sciences, Harvard University, Cambridge, 02138 USA; 4 National Oceanography Centre, Southampton, UK

**Keywords:** Climate change, Atmospheric science, Ocean sciences

## Abstract

Accurate historical records of Earth’s surface temperatures are central to climate research and policy development. Widely-used estimates based on instrumental measurements from land and sea are, however, not fully consistent at either global or regional scales. To address these challenges, we develop the Dynamically Consistent ENsemble of Temperature (DCENT), a 200-member ensemble of monthly surface temperature anomalies relative to the 1982–2014 climatology. Each DCENT member starts from 1850 and has a 5° × 5° resolution. DCENT leverages several updated or recently-developed approaches of data homogenization and bias adjustments: an optimized pairwise homogenization algorithm for identifying breakpoints in land surface air temperature records, a physics-informed inter-comparison method to adjust systematic offsets in sea-surface temperatures recorded by ships, and a coupled energy balance model to homogenize continental and marine records. Each approach was published individually, and this paper describes a combined approach and its application in developing a gridded analysis. A notable difference of DCENT relative to existing temperature estimates is a cooler baseline for 1850–1900 that implies greater historical warming.

## Background & Summary

Historical earth surface temperature estimates are important for monitoring climate change, attributing those changes to particular causes, and developing and communicating appropriate policy responses. Estimates of Earth’s surface temperature, however, involve a number of intricacies reflecting a heterogeneous observing network that is subject to various biases.

Land surface air temperatures (LSATs) are measured at approximately two meters above the ground, and LSAT records may contain discontinuities arising from changes in the types and exposure of thermometers, station relocation, and urbanization or other changes in the station environment^1^. Sea surface temperatures (SSTs) correspond to water temperatures of a layer that is generally within the top few meters of the ocean, though sometimes deeper^2^. Historically, SSTs were measured on traveling ships either by lowering buckets to collect water samples or as a by-product of monitoring engine room intake (ERI) temperatures^3^. Whereas bucket SSTs are mostly biased cold due to wind-induced evaporation, ERI SSTs are typically biased warm due to heat release from ship engines^4^. It was not until the 1990s that more accurate SST measurements became widely available in the form of observations from moored and drifting buoys^5^.

Accounting for these data biases, especially their changes over time, is crucial for quantifying and understanding long-term climate change. A variety of approaches exist for the homogenization and adjustment of temperature records over land^6,7^ and sea^8–12^. We briefly review existing datasets and the homogenization or adjustment methods used, and then outline certain improvements upon existing methods. These methodological improvements are used in constructing a new dataset called the Dynamically Consistent ENsemble of Temperature, or DCENT.

Some LSAT homogenization approaches rely upon national efforts conducted by some but not all countries, such as the Climate Research Unit Temperature (CRUTEM)^13^ and China Meteorological Administration datasets^14^. Another approach is to use global statistical homogenization algorithms, such as used in the Global Historic Climate Network (GHCNm)^7^ and Berkeley Earth Surface Temperature^15^. We recently described an extension to the homogenization algorithm proposed in ref. ^6^ to account for temporal auto-correlation in climate signals^16^, which is used to homogenize LSATs in DCENT.

The adjustment of ship-based SST measurements differs from that for LSATs because ships, unlike land-based weather stations, routinely move. Versions of NOAA’s Extended Reconstructed SST (ERSST)^11,12,17^ rely upon adjusting SSTs to be consistent with smoothed estimates of Nighttime Marine Air Temperatures (NMAT) from HadNMAT2^18^ on a global scale^11^. Other groups make use of the patterns of temperature biases expected on the basis of bucket models^8^, including COBE-SST2 from the Japanese Meteorological Agency^19^. For HadSST4 from the Met Office^10^, its SST correction before 1940 involves both adjusting SSTs to NMATs and using physical bucket models. DCENT takes a further step in the homogenization of ship observations by identifying offsets between different groupings of ships using pairwise comparisons of nearby observations^20^.

Beyond homogenizing LSATs and SSTs independently, there is also utility in ensuring physical and statistical consistency between these two domains of observations^21^. One means of homogenizing land and ocean temperatures is to compare and adjust records along coastlines. Ref. ^22^ linearly scaled coastal LSATs to infer near-coast SSTs and used the inferred coastal SSTs to adjust global SSTs. Building from ref. ^22^, we developed a coupled energy balance model (EBM) to better infer near-coast SSTs from coastal station temperatures. The model accounts for the physics of air-sea coupling and decreases the uncertainty of inferred near-coast SSTs, especially in the extra-tropics. Applying the coupled EBM to various versions of homogenized LSATs confirms the inconsistency between current LSAT and SST estimates on global and regional scales^23^. The homogenization between land and ocean temperature evolution is not included in presently available gridded datasets and is addressed in DCENT using the coupled EBM.

In the following we describe the development of DCENT, a land-ocean temperature dataset that is globally homogenized. DCENT takes advantage of recent methodological advances in temperature data homogenization, and is provided in the form of an ensemble of equally likely surface temperatures. We anticipate that DCENT, with its enhanced accuracy and comprehensive uncertainty estimation in data homogenization, to significantly advance our comprehension of historical warming.

## Methods

**Fig. 1:**
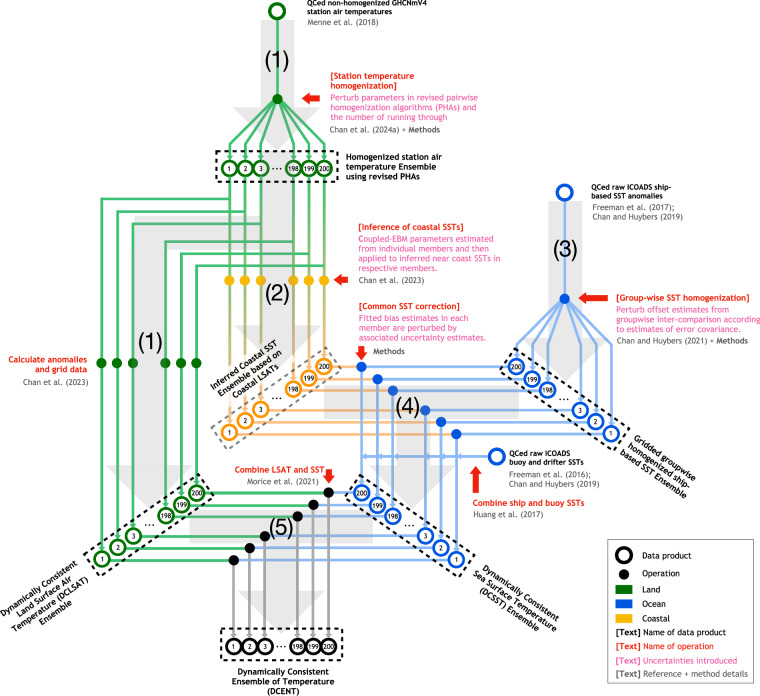
Five steps of DCENT development. A schematic for DCENT development, including (1) Homogenizing station temperatures and creating DCLSAT, (2) Inferring coastal SSTs from coastal land station temperatures, (3) Group-wise SST intercomparison, (4) Common SST adjustment, and (5) Combining DCLSAT and DCSST. Displayed are raw, intermediate and final data products (open circles and black text), processes and techniques used (solid circles and red texts), uncertainties accounted for (pink text), and references (gray text). Land air temperatures are in green, sea-surface temperatures are in blue, and temperatures along coast lines are in orange.

The development of DCENT involves five steps (Fig. 1). We first give an overview of these steps and then provide details in subsequent subsections.

1. **Homogenizing station temperatures and producing DCLSAT, the LSAT component of DCENT**: Starting from the unhomogenized monthly-resolution Global Historical Climatology Network version 4 (GHCNmV4)^7^, we apply pair-wise homogenization algorithms (PHA) to detect and adjust breakpoints. PHA compares nearby stations and detects breakpoints in inter-station differences using the Standard Normal Homogeneity Test^6^. Detected breakpoints are attributed to a particular station according to which station is most often associated with such a breakpoint when paired against a collection of other stations. We use two versions of PHA, both of which are recently revised to account for autocorrelation in climate variability^16^. The first version modifies the SNHT threshold to account for autocorrelation, and the second version replaces SNHT with a penalized likelihood (PL) approach wherein data is prewhitened using an order-one autoregressive model. These revised algorithms are more skillful than the existing, established algorithm^6^ in identifying the correct timing of breakpoints and recovering true climate variations^16^. More details regarding the revised algorithms can be found in Appendices B and C of ref. ^16^. Parameters in each algorithm are perturbed 50 times and combined to create a 100-member ensemble to quantify uncertainties associated with the timing and magnitude of the identified breakpoints^16^. An important issue with respect to homogenization approaches that depend on neighboring stations is that station coverage is sparse before the 20^th^ century. Existing pairwise algorithms may have insufficient neighbors for early stations, leading to incomplete adjustments in the late 19^th^ century. To account for data sparsity, we apply additional steps to each member of the ensemble created by ref. ^16^. These steps involve further iterations of PHA and breakpoint verification for stations with limited neighboring data. The ensembles after these additional steps are pooled together with the original ensemble to create a 200-member ensemble. Methodological details and implications for temperature reconstruction are discussed in sub-section M.1. Station-wise anomalies relative to the 1982–2014 climatology are calculated using a pairing and matching algorithm^23^ before binning to 5° × 5° monthly grids to create the 200-member DCLSAT ensemble.
2. **Inferring coastal SSTs from coastal land station temperatures**: In addition to gridding all land station records to create DCLSAT, we also use more than 3,000 coastal stations in each member of our pair-wise homogenized station temperature ensemble to infer near-coast SST evolution. This ensemble, named LSAT-inferred near-coast SST will be used for estimating SST adjustments (step 4). We use a coupled energy balance model (EBM) for land air and nearby sea surface temperatures, as detailed in ref. ^23^, for this inference. In short, the model uses land temperature and its tendency to predict the temporal evolution of nearby SSTs. The EBM implementation here is the same as ref. ^23^.
3. **Group-wise SST intercomparison**: Starting from releases 3.0.0 and 3.0.2 of the International Comprehensive Ocean-Atmosphere Data Set (ICOADS), we apply a group-wise intercomparison algorithm to homogenize ship-based SST measurements. ICOADS3.0.0 contains data until 2014, and ICOADS3.0.2 contains data from 2015 onward, allowing for extending the dataset to present. The pair-wise homogenization algorithms used for land temperatures are not directly applicable to marine measurements because ship motion leads to different recorders at any given point and hence different neighbors. A group-wise intercomparison method for homogenizing SSTs was first introduced in ref. ^24^ that extends the concept of pairing neighboring stations to pairing nearby SSTs from different groups. Groups are distinguished according to data source, measurement method, and countries, and a linear-mixed-effect model is used to estimate group-level offsets as a function of year and 17 sub-ocean basins. In ref. ^25^, the algorithm was run in a rolling manner, each comparing data in three consecutive months, in order to account for seasonality. In order to estimate the SST component for DCENT (DCSST), we revise the algorithm presented in ref. ^25^ to use a set of physics-informed patterns of bucket biases to account for seasonal and regional variations in group-wise SST offsets explicitly. Estimated offsets using the revised method hence have spatial and seasonal variations in line with physical expectations of how bucket-based SST measurements are biased. The revised method estimates offsets in all seasons simultaneously, giving fewer free-parameters and increased computational efficiency. Details of the physics-informed group-wise intercomparison method, as well as comparison with the existing approaches^25^, are in sub-section M.2. Groupwise offsets are randomly perturbed 200 times according to estimated uncertainties in order to create an intermediate ensemble of groupwise homogenized SSTs.
4. **Common SST adjustment**: The group-wise intercomparison method adjusts relative offsets but does not inform about biases common to all SST measurements. Because LSAT measurements are considered more reliable than SST measurements^21^, we use LSAT-inferred near-coast SSTs (step 2) to adjust group-wise homogenized observational SSTs, an idea similar to ref. ^22^. The common SST adjustment here assumes that SST biases estimated from coastal comparisons can be used to infer biases in the open ocean. Specifically, we use physically modeled patterns of bucket biases to estimate the spatial distribution of SST biases, anchoring these patterns to the coastal bias estimates. Details of this common SST adjustment are in sub-section M.3. To propagate the uncertainty of land temperature estimates, each member of the LSAT-inferred near-coast SST ensemble is randomly paired with a groupwise homogenized SST member without repetition. The adjusted ship-based SST ensemble is then combined with moored buoy and drifter SST anomalies to generate the gridded DCSST product.
5. **Combining DCLSAT and DCSST**: Finally, each DCLSAT member is combined with its DCSST member pair from the common SST adjustment (step 4) to create the dynamically consistent ensemble of temperature (DCENT). Over coastal regions where both LSAT and SST are available, the combination is weighted by the fraction of land and ocean area in individual grid boxes, as provided by ref. ^26^. When either LSAT or SST is available, the weight of the available component is one. Moreover, the ensemble contains comprehensive uncertainty quantification for the adjustments applied, including land station homogenization, the inference of coastal SSTs, groupwise SST adjustments, and common SST adjustments.

### Updates in land station temperature homogenization

**Fig. 2:**
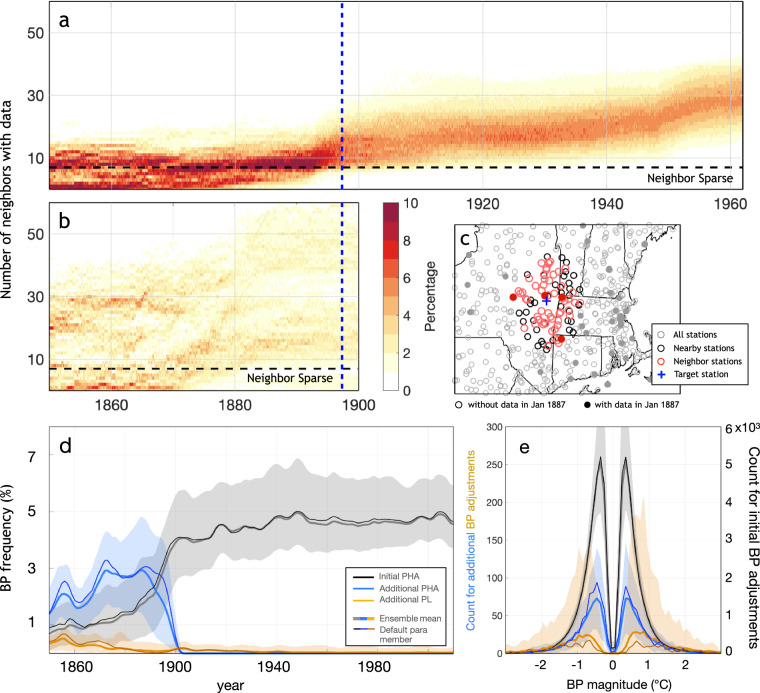
Land temperature homogenization in the 19^th^ century. (**a**) Density plot of the number of neighbors with data (y-axis) as a function of time (x-axis). Results are shown for the default parameter combination of the pairwise homogenize algorithm (PHA) in ref. ^16^. (**b**) as panel (**a**) but for a second round of group-wise homogenization using only stations having data before 1900, which effectively increases the search radius for neighbors and hence reduces the number of neighbor-sparse stations. (**c**) station network in the Northeastern US. Markers shows a target station (blue cross), the nearest 100 stations (black), 40 neighboring stations (red), and other stations (gray). Stations with valid data reported in 1887 are filled circles, and otherwise open circles. (**d**) 11-year running averaged frequency of detecting breakpoints as a function of years for the ensemble in ref. ^16^ (black). Also shown are results after running an additional PHA for stations having data in the late 19^th^ century (blue), and after running penalized likelihood for stations that remain data sparse after two rounds of PHA (orange). Shown results are for the default parameter combination (thin curves), mean over the 100-member ensemble (thick curves), and the 95% confidence interval (c.i., shading). (**e**) as (**d**) but for the histogram of breakpoint magnitudes. Note that the left and right y-axes have different scales.

Ref. ^16^ introduced two revisions to a benchmark pair-wise homogenization algorithm (PHA) by accounting for auto-correlation in breakpoint detection. Improvements in the accuracy of identified breakpoints and recovered underlying climate variability has been illustrated using synthetic data, climate model simulations, and comparisons against available historical metadata^16^. That said, station coverage was sparse before the 20^th^ century and has led to an apparent decline in the percentage of station with sufficient neighbors for pair-wise comparison in the later half of the 19^th^ century (Fig. 2a). An example could be the station network in the Northeastern US (Fig. 2c). Around the target station in New Hampshire (blue cross), PHA first pre-selects the nearest 100 stations (black) and of those selects the 40 stations best correlated with the target (red circles). However, among the initial 100 stations, many do not have data that extends back to the late 19^th^ century (open circles). Stations with data from that period (filled circles) are often too far away to be considered using the modern station network. Consequently, the frequency of effective comparisons against neighbors decreases significantly before 1900, leading to a reduction in the detection and adjustment of breakpoints (black lines in Fig. 2d). This issue potentially results in insufficient adjustments for the period before the 20^th^ century.

We performed two additional analyses to account for the sparsity of early stations. First, after performing a first round of PHA, we subset all stations that have data before 1900 and run a second round of PHA on homogenized pre-1900 data from this station subset. Such a practice effectively uses a larger radius for neighbor-selection and increases the number of valid neighbors substantially (Fig. 2b). Around 2-3% more breakpoints are identified before 1897 (blue curves in Fig. 2d), suggesting that the rate of breakpoints in station records could be more consistent over time than previously suggested^16^.

**Fig. 3:**
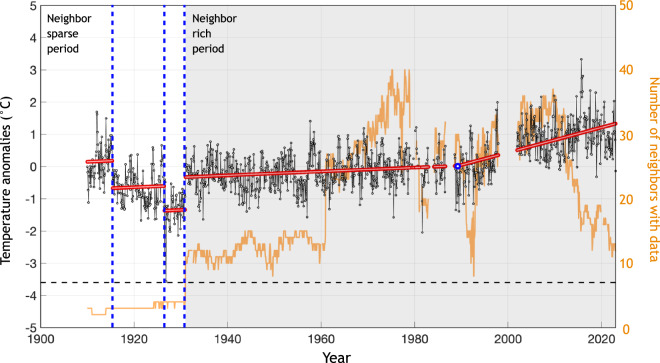
An illustration of applying the penalized likelihood method in producing the DCLSAT ensemble. This station has an insufficient number of valid neighbors before the 1930s (orange curve) after running two rounds of PHA. A penalized likelihood method is hence running on temperature record from this station alone (black curve) to detect potential discontinuities in records. Our model allows for detecting discontinuities in mean (dashed vertical blue lines in 1916, 1926, and 1931) and trend (blue circle in 1988) separately. The red line shows the best fit.

After two rounds of PHA, some stations still have insufficient valid neighbors, at least during some periods. Among the ~28,000 stations in GHCNmv4, the number of these isolated stations ranges from 138 to 2439 across the 100-member ensemble. This variation reflects different parameters, such as the number of initial stations checked for identifying neighbors and the definition of data sparsity used across ensemble members. We, therefore, also perform a separate check for each of these stations for breakpoints during its neighbor-sparse period. Specifically, we use a penalized likelihood approach similar to that used in ref. ^27^. Because we are not comparing isolated stations against neighbors, and the actual climate variability could be large, we use a more flexible model setup that allows for placing change points in mean and trend at different times, i.e.,

1 $$T=\mu +kt+\mathop{\sum }\limits_{i=1}^{\mathop{\max }\limits_{j}(t > {t}_{j})}\Delta {\mu }_{i}+\mathop{\sum }\limits_{i=1}^{\mathop{\max }\limits_{j}(t > {\tau }_{j})}(t-{\tau }_{j})\Delta {k}_{i}+{\varepsilon }_{t},$$

where *μ* and *k* are the mean and trend before the first change point. List *t*_*j*_, *j* = 1, 2, …, *m* denotes the timing of *m* breakpoints with the magnitude relative to the previous segment denoted as Δ*μ*_*j*_, and list *τ*_*j*_, *j* = 1, 2, …, *n* denotes the timing of *n* change points in trend with the magnitude of trend change relative to the previous segment denoted as Δ*k*_*j*_. In the current setup, *t*_*j*_ can only take values in data sparse periods. Term *ε*_*t*_ is the error process, representing natural temperature fluctuations, and is modeled as an AR(1) process. The penalized likelihood of Eq. (1) is,

2 $$T=N\log ({\widehat{\sigma }}^{2})+N+N\log (2\pi )+(2m+2n+4)\log (N),$$

where *N* is the length of the series, and ${\widehat{{\rm{\sigma }}}}^{2}$ is the residual of an ordinary least square fit. We use a multi-parent genetic algorithm to optimize Eq. (2) (see Appendix C in ref. ^16^ for detailed implementation). An illustration of station record, data sparse period, and fitted change points are in Fig. 3. This double-check based on penalized likelihood identifies most breakpoints before 1880 (Fig. 2d), a period during which a considerable portion of stations are still data-sparse in the second round of PHA (Fig. 2b).

Compared with only running the first step of PHA, these additional steps, on average, revise temperature over 1850–1900 to be 0.03 [0.00,0.06] °C cooler, suggesting a slightly higher degree of warming since the pre-industrial period.

### Physics-informed group-wise SST intercomparison

**Fig. 4:**
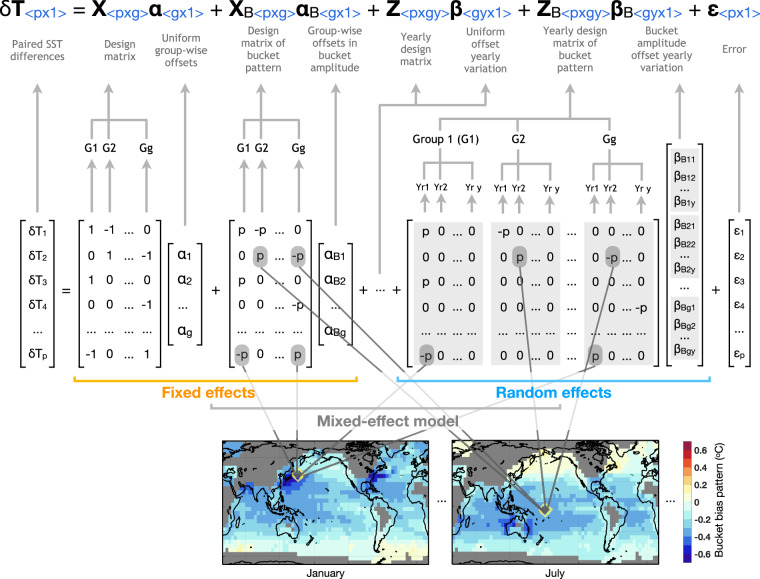
An element-wise illustration of the linear-mixed-effect model for the physics-informed group-wise intercomparison. Equation (3) is given, together with the dimensions of matrices and vectors (blue), where p, g, and y are, respectively, numbers of pairs, groups, and 5-yr increments, respectively. Four terms are illustrated in detail: (1) paired SST differences *δ*T; (2) *X* is a design matrix that specifies group-wise interactions between paired observations, and *α* represents the fixed effects of global and seasonal mean offsets; (3) *X*_*B*_ is a design matrix that specifies not only groups but also the monthly bucket bias pattern where comparisons occur (*p* and − *p*), and *α*_*B*_ represents the fixed effects of group-wise offsets congruent with bucket patterns; and (4) *Z*_*B*_ is a design matrix expanded to specify 5-yr bins in which group-wise comparison take place, and *β*_*B*_ represents 5-yr random effects of bucket magnitude that are assumed to follow a Gaussian distribution. Bucket patterns are resolved at 5° × 5° monthly resolution, and the two snapshots in January and July are shown as an illustration.

We inform the group-wise intercomparison algorithm with simulated monthly patterns of physically-based bucket biases to explicitly account for seasonal and regional variations in group-wise SST offsets. Specifically, we use the canvas bucket model in ref. ^28^. Bias patterns, **P**, are resolved at monthly 5° resolution, and examples of January and July patterns are in Fig. 4. Using the physical patterns of biases expected on the basis of bucket models reduces the number of parameters by a factor of 9 and allows for better quantification of uncertainties. Specifically, we specify a linear mixed effect model,

3 $$\delta {\bf{T}}={\bf{X}}{\rm{\alpha }}+{{\bf{X}}}_{{\rm{B}}}{{\rm{\alpha }}}_{{\rm{B}}}+{\bf{Z}}\beta +{{\bf{Z}}}_{{\rm{B}}}{\beta }_{{\rm{B}}}+\varepsilon ,$$

where *δ***T** is a vector of SST differences within individual pairs. **X** is a design matrix, whose entries are 0, 1, and -1, to indicate which two groups are being compared. *α* is the fixed effect and contains systematic offsets for individual groups. **Z** is another design matrix similar to **X** but also indicates in which 5-year increment comparisons occur. *β* is the random effect and contains variations of offsets across 5-year increments for individual groups. *ε* denotes random error. These terms are identical to ref. ^25^. On the other hand, **X**_B_α_B_ and **Z**_B_*β*_B_ are terms associated with bucket patterns. **X**_B_ is a design matrix whose entries are 0, *p*, and − *p*, where *p*(*ϕ*, *θ*, *m*) denotes bucket SST bias patterns sampled from **P** at longitude *ϕ*, latitude, *θ*, and calendar month, *m*. Accordingly, α_B_ denotes mean groupwise offsets in the magnitude of bucket patterns. Similarly, *β*_B_ denotes temporal variations in the magnitude of the bucket pattern, and **Z**_B_ is the associated design matrix. An element-wise visual representation detailing the construction of this physics-informed linear-mixed effect model is in Fig. 4.

We use the revised groupwise intercomparison method to homogenize all ship-based SST measurements in ICOADS3.0.0 and 3.0.2^29^, where ICOADS3.0.2 contains data from 2015 to 2023. The identification of ship-based SSTs, initial quality control, and group assignment are identical to ref. ^25^. The current analysis contains ~50 million pairs coming from 513 groups that each contributes to at least 5,000 SST pairs. To increase computation efficiency, we aggregate data by averaging SST differences and design matrices according to combinations of pairs of groups, 5-year increments, calendar month, and 30° × 15° longitude-latitude boxes, which reduces the number of pairs to 4.2 million.

### Common SST adjustment using near-coast SSTs inferred from coastal weather stations

Inferred coastal SSTs are paired with group-wise homogenized SSTs for estimating common SST biases after the 1930s, when the SST archive is a mixture of bucket and engine-room-intake (ERI) measurements, using the following linear model (Model 1),

4 $$\delta {\bf{T}}=\gamma +{{\bf{X}}}_{{\rm{B}}}{\gamma }_{{\rm{B}}}+\varepsilon .$$

Vector *δ***T** contains the difference between group-wise homogenized and inferred coastal SSTs binned to monthly 5° resolution. The elements of vector **X**_B_ are sampled from the bucket bias patterns according to longitude, latitude, and calendar month. Term *γ* is similar to *α* in Eq. (3) and denotes the spatially and seasonally invariant biases, representing those congruent with ERI measurements, which have been assumed to be less influenced by environmental factors^9^. Term *γ*_*B*_, similar to *α*_*B*_ in Eq. (3), denotes the magnitude of common biases that can be projected onto the bucket pattern.

Before 1930, the SST archive contains mainly bucket measurements^20^, and we use a model that only projects onto the bucket patterns to estimate required adjustments (Model 2), i.e.,

5 $$\delta {\bf{T}}={{\bf{X}}}_{{\rm{B}}}{\gamma }_{{\rm{B}}}+\varepsilon .$$

We assume a linear transition from using Model 2 to Model 1, with the starting and ending years of the transition randomly drawn from uniform distributions *U* (1907, 1913) and *U* (1927, 1933), respectively.

The solution of both models are found using weighted least squares, with the weight being the inverse of the uncertainty in each monthly 5° box. The uncertainty model is, ${\sigma }_{\epsilon }^{2}={\sigma }_{s}^{2}+{\sigma }_{r}^{2}+{\sigma }_{c}^{2}+{\sigma }_{i}^{2}+{\sigma }_{l}^{2}/{n}_{l}$. Terms ${\sigma }_{s}^{2}$, ${\sigma }_{r}^{2}$ and ${\sigma }_{c}^{2}$ denote SST’s sampling uncertainty, random observation uncertainty and systematic ship-level uncertainties, respectively. We use the HadSST4 estimates for these values^10^. Term ${\sigma }_{i}^{2}$ denotes the uncertainty of inferred coastal SSTs and is estimated to have a spatially averaged standard error of 0.36°C at monthly 5° grid level^23^. Term ${\sigma }_{l}^{2}$ denotes the uncertainty of monthly station temperatures, which we assume to decrease with the number of stations in a grid (*n*_*l*_), and we estimate its value (0.41°C; 1 s.d.) using island grids in CRUTEM5^13^.

**Fig. 5:**
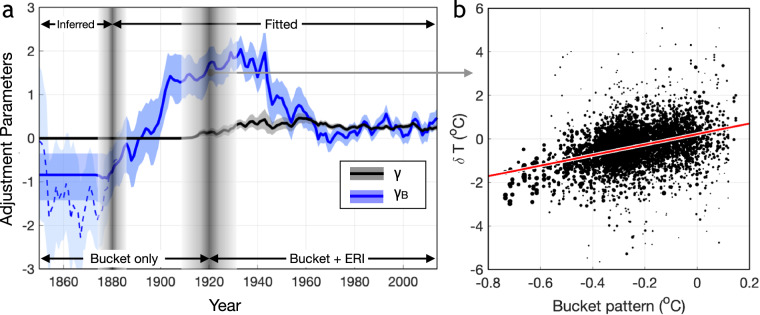
Common SST bias adjustment. (**a**) Estimated magnitude of biases congruent with bucket patterns (blue) and globally and seasonally uniform bias (black). Shading denotes 95% c.i. across the 200 DCSST members. Whereas values after 1880 are estimated from comparing LSAT-inferred versus group-wise adjusted observational SSTs along global coasts, those before 1880 are assumed to have the same value because early coastal land stations are sparse (ref. ^23^). The dashed line shows the result if we estimate bias using pre-1880 data. Moreover, SST measurements are primarily from bucket before 1930 but consist of bucket and engine-room-intake measurements afterwards. To account for this distinction, we fit two models, using bucket pattern plus an intercept (Model 1) and the other with only the bucket pattern (Model 2). The transitions between using inferred to fitted biases and using Model 2 to Model 1 are indicated by gray vertical shadings. (**b**) The difference between group-wise adjusted minus LSAT-inferred coastal SSTs (y-axis) scales positively with collocated bucket bias patterns (x-axis). Data shown are for 1920. Each marker is a 5° × 5° monthly box with size indicating number of contributing SST observations. Also shown is a linear fit using ordinary least squares (red line).

Moreover, ref. ^23^ concluded that the sparsity of land stations would result in the standard error of averaged coastal SSTs inferred from station temperature higher than 0.05°C. In addition, the group-wise SST intercomparison could be incomplete due to limited metadata available for grouping in the early period, introducing another layer of complexity. As a result, we use the estimated common bias in 1880 to adjust SSTs over 1850–1880, a practice that has been adopted in producing other SST products^12^. In other words, the common SST bias adjustment in DCSST involves adjusting inferred bias estimates from 1850–1880, using biases fitted from a bucket-pattern only model until the 1910s, linearly transitioning to biases fitted from a bucket-pattern plus intercept model until the 1930s, and continue all the way to present (Fig. 5a).

Removing the estimated common SST biases leads to our fully adjusted ship-based SSTs, which are then merged with data from both moored and drifting buoys, identified using ICOADS ID indicator, source ID, deck, and platform metadata^25^. Observations from buoy and drifter SSTs begin in the early 1980s. Notably, these measurements are typically 0.1°C warmer than the OISST reference used for the anomaly calculations. This results in a noticeable mean offset between the ship-adjusted data and buoy/drifter SSTs. To address this offset, we adopt the approach detailed in ref. ^17^, removing the average 1982–2022 discrepancies between buoy and adjusted ship-based SST anomalies from buoy SSTs. These adjustments are made at a monthly 5° resolution. Leveraging insights from ref. ^17^, we assign a weight to buoy and drifter SSTs that is 6.8 times of ship-based SSTs, where the weighting is determined by comparing their uncertainty estimates.

## Data Records

This data descriptor presents version 1.0 of DCENT, which is available as 200 NetCDF files named in the format of “DCENT_ensemble_1850_[YYYY]_member_[XXX].nc”, via HarvardDataverse^30^ (10.7910/DVN/NU4UGW). The variables in individual files are described in Table 1. In addition, the ensemble mean is provided in “DCENT_ensemble _1850_[YYYY]_ensemble_mean.nc”, and monthly climatological temperatures at 5° × 5° resolution are available in “DCENT_monthly_climatology_1982_2014.nc” to facilitate the determination of absolute temperatures. To support users requiring access through cloud computing, DCENT is also accessible in .zarr format in the Google Cloud bucket “dcent_dynamically_consistent_ensemble _of_temperature”. A Jupyter notebook script for accessing DCENT on Google Cloud is available at 10.7910/DVN/NU4UGW.

**Table 1:** Details of variables provided in DCENT.

Variable	Long name	Description
lon	Longitude	Longitude of the center of 5° cell (2.5°E–357.5°E)
lat	Latitude	Latitude of the center of 5° cell (−87.5°S–87.5°N)
time	Time	Midpoint of a month (days since Jan. 1^st^, 1850)
temperature	Temperature anomaly	Combined LSAT and SST anomalies relative to the 1982–2014 mean (in °C)
sst	Sea surface temperature anomaly	SST anomalies relative to the 1982–2014 mean (in °C)
lsat	Land surface air temperature anomaly	LSAT anomalies relative to the 1982–2014 mean (in °C)

## Technical Validation

### Summary of methodology evaluation

The methodologies employed in DCENT development have undergone thorough evaluation across different aspects.

1. **Validation of revised pair-wise homogenization algorithms for land station temperatures**: The benchmark pair-wise homogenization algorithm, which doesn’t consider auto-correlation, has been shown to be skillful in recovering long-term trends^6,31^. This validation has been conducted using synthetic data, simulated temperatures, and reanalysis products with introduced breakpoints, either randomly introduced or clustered in time^6,31^. In direct comparison with the benchmark algorithm, the algorithms used in DCENT demonstrate superior performance across various metrics. When tested on synthetic data and also the Coupled Model Intercomparison Phase 6 (CMIP6) simulations with randomly introduced breakpoints, they correctly identify more breakpoints, making fewer false identifications, and showing lower root mean square error (RMSE) in the recovery of long-term trends^16^. Furthermore, the validation process included a comparison with station metadata from the Historical Observing Metadata Repository^16^. When revised pairwise homogenization algorithms are applied to GHCNmV4 data, the percentage of identified breakpoints matching with metadata indicating station re-locations and instrumental changes was found to be 14% and 10% higher (p < 0.01) compared to random breakpoint identification. These percentages were also 1% higher (p < 0.1) than those achieved by the benchmark algorithm, highlighting improved skill of the revised algorithms. To further evaluate DCENT land temperature estimates, we compare the datasets against a tree-ring-based temperature reconstruction^32^ following the approach of ref. ^33^. The proxy-based temperatures suggest cooler summertime land temperatures during the late 19th century, and they show the highest consistency with DCENT land temperatures in the baseline period compared to other instrumental datasets (Fig. 6).

**Fig. 6:**
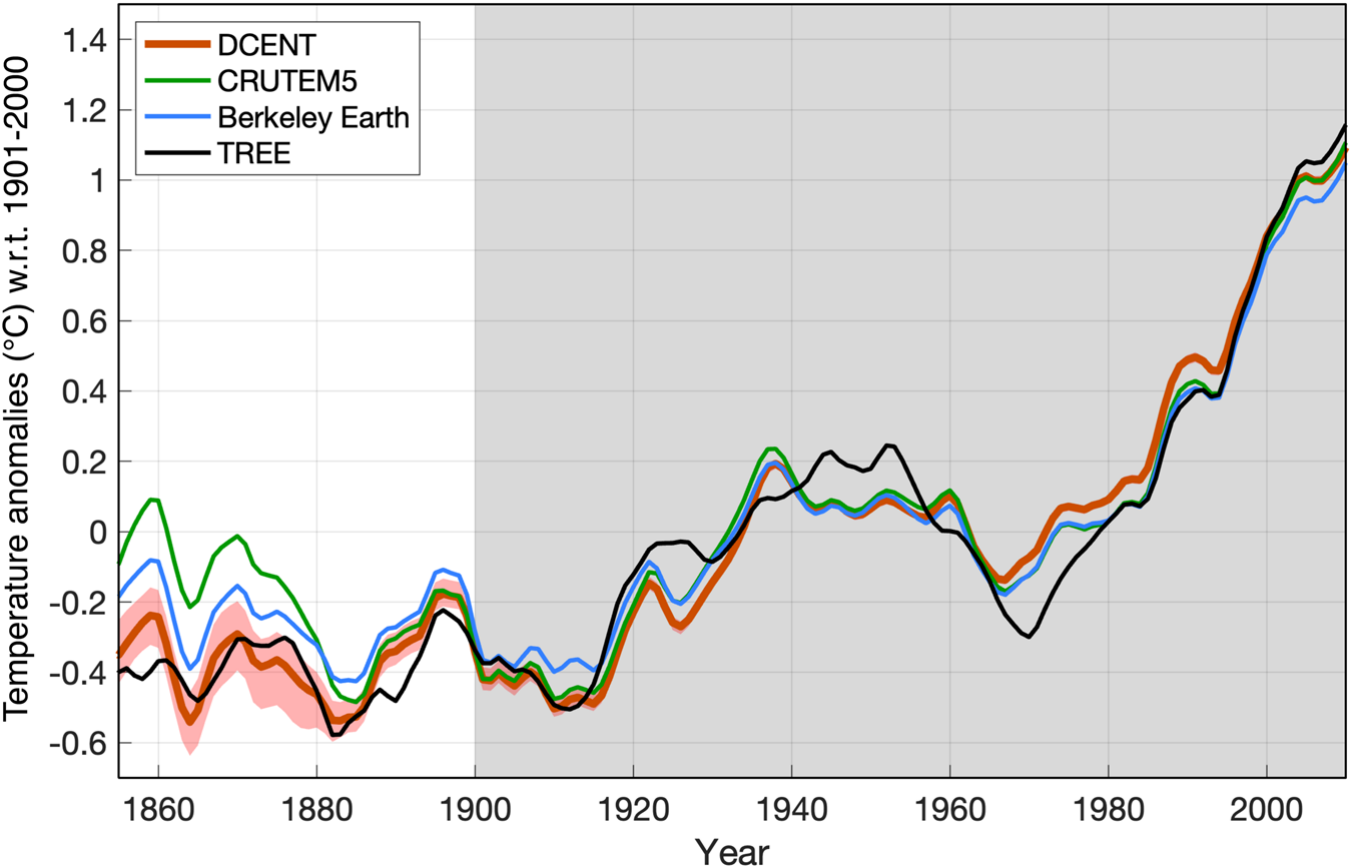
Comparison of instrumental and tree-ring-based temperature reconstructions. Land air temperature anomalies for the Northern Hemisphere growing season (May-August) are compared across instrumental datasets – DCENT (red), CRUTEM5 (green), and Berkeley Earth (blue) – and a tree-ring-based temperature reconstruction (black). Anomalies are calculated relative to the 1900-2000 mean, and the tree-ring-based temperatures are re-scaled to have the same interannual variance as the instrumental temperatures following the methodology of ref. ^33^. The comparison is performed using the least common coverage over the Northern Hemisphere (20° N poleward) across all datasets to ensure a fair evaluation.
2. **Validation of the physics-informed group-wise intercomparison algorithm for SSTs**: The validation of the benchmark algorithm^25^, which doesn’t account for physically simulated patterns of bucket SST biases, has been conducted using both physical and historical evidence. Identified group-wise offsets across groups of bucket measurements are negatively correlated with the amplitude of the SST diurnal cycle^28^. This relationship aligns with the physical expectation of inaccurate metadata categorizing engine-room-intake (ERI) measurements as buckets. ERI measurements exhibit a warm bias due to engine heat but have small diurnal cycles since they sample at depths of 5-15m, which are less influenced by daily temperature fluctuations^4,34^. Removing estimated offsets between groups of ERI and bucket SSTs suggests no apparent SST warming during the World War II period, consistent with contemporary land temperature evolution^25^. Moreover, the group-wise intercomparison algorithm also identified a sudden drop of around 0.4° C in SSTs from the Japanese KOBE Collection relative to other groups, specifically in the 1930s^20^. This finding is consistent with a truncation error that floored all temperature measurements from the KOBE collection to whole degrees Celsius since the 1930s^35^. Adjusting for this bias resulted in continuous early-20^th^-century warming, whose magnitude is comparable to the North Atlantic warming, over the North Pacific^20^. Updating the groupwise intercomparison using bucket patterns yields highly consistent global and basin-scale adjustments (Fig. 7j–l). Moreover, it prevents apparent boundaries of SST adjustments in the open ocean (Fig. 7a–i).

**Fig. 7:**
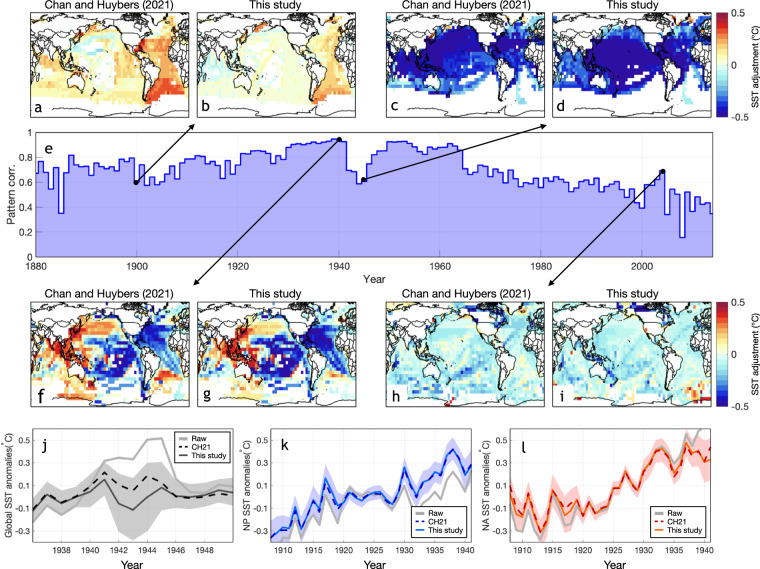
Comparison of group-wise SST adjustments between ref. ^25^ and DCSST. (**a**) the pattern of 1900 group-wise SST adjustments in ref. ^25^. Note the apparent boundaries in the open ocean due to estimating regional variations of offsets using 17 sub-basins. (**b**) as (**a**) but for adjustments from this study. Using bucket patterns prevents the existence of apparent boundaries. (**c–d**) as (**a–b**) but for year 1945. (**e**) Pattern correlation of annual group-wise adjustments between ref. ^25^ and this study. The group-wise comparison in this study has nine times fewer parameters but the pattern correlation remains higher than 0.6 throughout 1880–2000. (**f–g**) as (**a–b**) but for year 1940. (**h–i**) also as (**a–b**) but for year 2005. (**j-l**) group-wise adjustments in both ref. ^25^ (dashed) and this study (solid) yield highly consistent results in removing SST biases at global and regional scales, including abnormally warm SSTs during World War 2 (**j**), cold truncation bias in Japanese measurements after the 1930s over the North Pacific (**k**), and warm bias associated with engine heat release after the 1930s over the North Atlantic (**l**).
3. **Validation of inferring coastal SSTs from nearby LSATs**: The coupled EBM has been shown to capture different regimes of air-sea interaction^23^. The spatial distribution of model parameters derived from recent observations are also consistent with those fitted from CMIP6 simulations and are aligned with physical expectations^23^. The EBM parameters have also been shown to be quasi-stationary throughout the historical period by comparing CMIP6 results during the first and second halves of the 20^th^ century^23^. Compared with an earlier method that linearly scales LSAT anomalies to infer coastal SSTs^22^, using the coupled EBM significantly reduces inference error, especially for the extra-tropics during wintertime^23^.
4. **Validation of common SST adjustment**: The difference between LSAT-inferred and groupwise homogenized coastal SSTs show an apparent positive correlation with the assigned bucket bias pattern before the 1940s (p<0.01, Fig. 5b). Moreover, DCENT’s common SST adjustment show a rapid increase from 1880 to 1900 (Fig. 5a), which is consistent with a rapid increase in diurnal amplitude and the physical expectation of using a less insulated bucket^25^. In addition, both DCENT’s common SST adjustment and the diurnal amplitude evolution can be simulated physically using bucket models representing different bucket sizes and levels of insulation^28^.

### Comparison with other data products

**Fig. 8:**
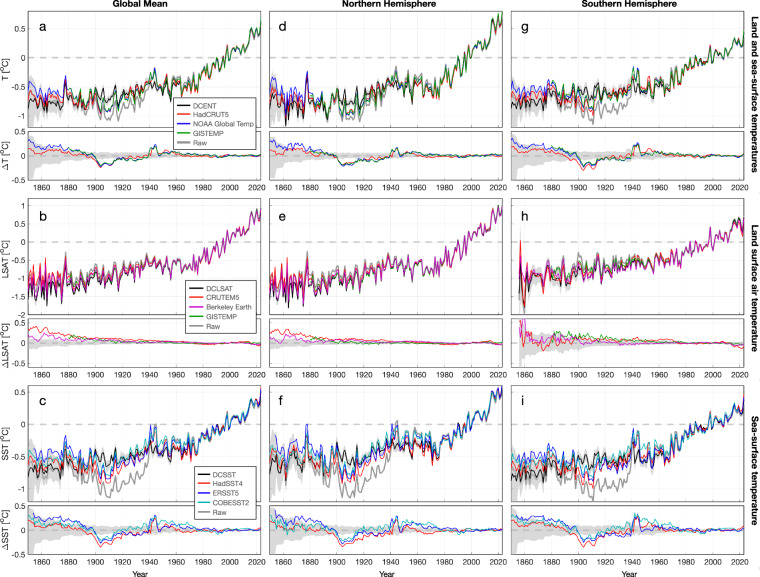
Comparisons of temperature estimates on global and hemispheric scales. (**a**) Upper: annual global mean surface temperature anomalies from DCENT (black), HadCRUT5 (red), NOAA global temperature (blue), GISTEMP (green), and unadjusted records (gray). Shading denotes 95% c.i. for the 200-member DCENT ensemble. Lower: difference relative to the ensemble mean estimate of DCENT. Anomalies are relative to the 1982–2014 mean, and all datasets are regridded to 5° resolution and reduced to the least common coverage. (**b**) as (**a**) but for continental mean land air temperature estimates from DCLSAT (black), CRUTEM5 (red), Berkeley Earth (purple), and GISTEMP (green). (**c**) as (**a**) but for global mean sea-surface temperature estimates DCSST (black), HadSST4 (red), ERSST5 (blue), and COBE-SST2 (cyan). (**d-f**) and (**g-i**) as (**a-c**) but for the Northern Hemisphere and the Southern Hemisphere, respectively.

**Fig. 9:**
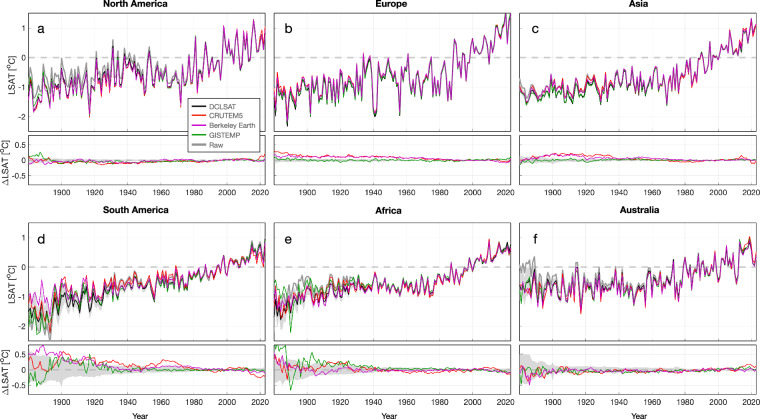
Comparison of regional land surface air temperatures. Individual panels are as (**b**) in Fig. 8 but for (**a**) North America, (**b**) Europe, (**c**) Asia, (**d**) South America, (**e**) Africa, and (**f**) Australia.

**Fig. 10:**
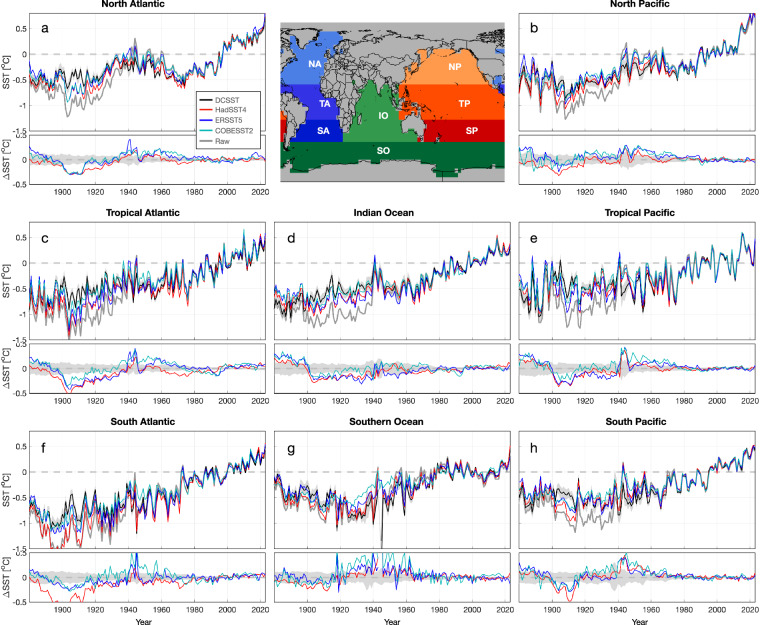
Comparison of regional sea-surface temperatures. Individual panels are as (**c**) in Fig. 8 but for (**a**) the North Atlantic, (**b**) the North Pacific, (**c**) the tropical Atlantic, (**d**) the Indian Ocean, (**e**) the Tropical Pacific, (**f**) the South Atlantic, (**g**) the Southern Ocean, and (**h**) the South Pacific.

When compared to existing estimates, DCENT demonstrates a high level of consistency at global, hemispheric, and regional scales for both land and sea-surface temperatures after the 1960s (Figs. 8, 9, 10). Before the 1960s, DCENT global and hemispheric mean temperatures show lower temperatures during World War II (WWII), systematically higher temperatures from 1900 to 1940, and lower temperatures over 1850–1890 (Fig. 8a,d,g). Specifically, the warm anomaly during WWII and the cold anomaly over 1900–1910 in other existing estimates (HadCRUT5 ^26^, NOAA Global Temp^36^, and GISTEMP^37^) falls outside the range of the DCENT ensemble. In contrast, the difference during the late 19^th^ century remains within uncertainty estimates.

Looking at the land and the ocean separately indicates the source of discrepancies between DCENT and other products. Over land, all temperature estimates show a consistent pattern, with warming from 1850 to 1940, followed by a period of warming hiatus over 1940–1970, and a rapid warming until present (Fig. 8b,e,h). Despite this overall consistency, DCLSAT indicates colder temperatures from 1850 to 1900 than all other estimates. Whereas Berkeley Earth^15^ and GISTEMP^37^ estimates are on the upper bound of the DCENT ensemble, CRUTEM5^13^ falls outside the DCENT range, especially over the Northern hemisphere. Note that, unlike all other estimates, CRUTEM5 does not homogenize station temperatures using a global homogenization algorithm^13^. On the other hand, DCENT runs revised global homogenization algorithms twice and hence is likely to remove the influence of breakpoints more completely, especially in the Northern Hemisphere where the station density is higher.

Not surprisingly, the most substantial difference is in the SST component. DCSST is systematically cooler than all other estimates in the late 19^th^ century but becomes systematically warmer in the early 20^th^ century (Fig. 8c,f,i). From 1850 to 1900, whereas HadSST4^10^ and COBE-SST2^19^ falls in the 95% c.i. of DCSST, ERSST5^12^ is significantly warmer than DCSST. During the early 20^th^ century, especially between 1900–1920, all other estimates are colder and fall outside the range of DCSST. These differences are consistent between hemispheres (Fig. 8f,i). This difference is due to the different approach used in DCSST to adjust early SST biases. Whereas DCSST was referenced against coastal station temperatures, ERSST5^12^ and HadSST4^10^ were referenced to night-time marine air temperatures (NMAT). The contrast between DCSST and other estimates also implies that coastal air temperatures do not align with NMATs before the 1940s, a discrepancy reported in ref. ^38^. Although NMAT may entail biases due to variations in ship height^18^, the underlying cause of the difference between coastal-LSAT and NMAT references remains elusive. Nevertheless, this comparison underscores the inconsistency between LSATs and NMATs and indicates that further investigation of this divergence is warranted.

Further breaking-down to regional temperatures, DCLSAT is largely consistent with other estimates over North America, Europe, Asia, and Australia (Fig. 9a–c,f). South America and Africa show different regional variations, however, especially before the 1920s. DCLSAT suggests significantly cooler South American temperatures in the late 19^th^ century than the Berkeley Earth estimate (Fig. 9d). That said, both estimates suggest a continuous warming throughout 1880 to 1940, whereas in CRUTEM5 and GISTEMP, South American temperatures contain an apparent discontinuity in the late 1890s. For African temperatures before the 1940s, whereas DCLSAT, CRUTEM5, and Berkeley Earth estimates are consistent within uncertainties, GISTEMP suggests a significantly warmer African temperature in the 1880s. For regional SSTs, the difference between DCSST and other estimates is generally consistent with that on the global scale (Fig. 10).

**Fig. 11:**
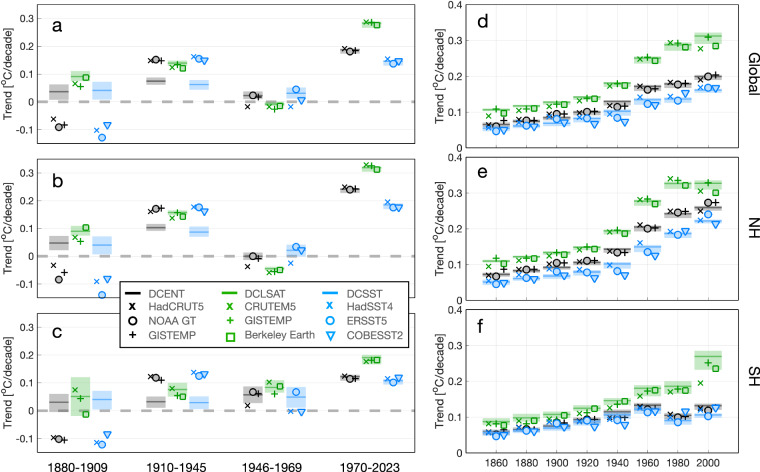
Comparison of decadal and long-term trends of global and hemispheric temperatures. (**a**) Decadal temperature estimates over individual decades (x-axis) for combined land-sea temperature estimates (black), LSATs (green), and SSTs (blue). The bars and shading denote DCENT/DCLSAT/DCSST estimates with the 95% c.i., and markers denotes other estimates as indicated in the legend. (**b–c**) are as (**a**) but for the Northern Hemisphere and the Southern Hemisphere, respectively. (**d–f**) as (**a–c**) but for long-term trends ending in 2023, with starting year indicated in the x-axes.

**Fig. 12:**
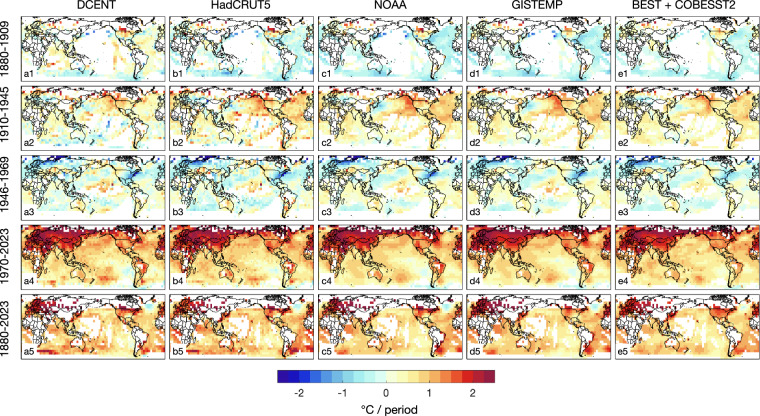
Comparison of the pattern of decadal and long-term trends. (a1–a5) DCENT decadal trends over (a1) 1880–1909, (a2) 1910–1945, (a3) 1946–1969, (a4) 1970–2023, and (a5) 1880–2023. For trends to be estimated in a grid cell, we require at least half of the decades, together with the first and the last decade, to have valid data coverage. For a decade to be valid, at least five years of data should each has at least six month being sampled. Columns b–e are as (**a**) but for other estimates from (**b**) HadCRUT5, (**c**) NOAA global temperature, (**d**) GISTEMP, and (**e**) combining Berkeley Earth over land and COBE-SST2 over the ocean (for display purposes). The combination of Berkeley Earth LSAT and COBE-SST2 involves replacing empty grid cells in Berkeley LSAT trends with COBE-SST2 trends where-ever possible. Before calculating trends, all datasets are regridded to 5° × 5° spatial resolution and reduced to the least common coverage, or those grid boxes containing data across all products.

Next we compare decadal trends by dividing the temperature record into four intervals: 1880–1909, 1910–1945, 1946–1969, and 1970–2023, based on the evolving patterns of warming, cooling, or neutral trend. During the 1970–2023 interval, DCENT’s trends exhibit remarkable consistency with other temperature estimates on global and hemispheric scales for both LSATs and SSTs (Fig. 11a,b,c). The spatial distribution of warming trends is highly congruent across the different estimates (Fig. 12a4–e4), depicting intensified warming over land, particularly prominent over the Eurasian landmass. Over the ocean, SST trends since the 1970s reveal a La Niña-like pattern. While COBE-SST2 does not portray evident cooling in the Eastern South Pacific, similar to other estimates, all datasets indicate greater warming in the Western Equatorial Pacific warm pool region than in the Eastern Equatorial Pacific.

In the period from 1946 to 1969, the global mean temperature displays modest trends and regional patterns of warming again closely align, especially among the estimates from DCENT, NOAA, and GISTEMP (Fig. 11a,b,c). This congruence is consistently observed when examining trends within LSAT and SST estimates. Features include weak warming in the tropical oceans and cooling in regions such as the Eastern United States, the Mid-latitude North Atlantic, Europe, and Russia (Fig. 12a3–e3).

Substantial trend discrepancies emerge before 1945. In the early 20^th^ century (1910–1945), all other estimates exhibit significantly faster warming trends than all 200 members of DCENT on both global and hemispheric scales (Fig. 11a,b,c). This discrepancy primarily stems from differences in SST estimates, whereas LSAT trends remain consistent between DCLSAT and other estimates. It is worth noting that the 1910–1945 SST warming in estimates other than from DCSST surpass contemporary land warming and SST warming in certain regions over recent decades. DCSST mitigates this rapid warming on account of warmer SSTs in the 1900s as well as removing the World War II warm anomaly following the group-wise intercomparison^25^ (Figs. 8 and 10).

During the late 19^th^ century (1880–1909), all LSAT estimates indicate warming, but SST estimates in datasets other than DCSST show broadly uniform cooling (Fig. 11a,b,c). In contrast, DCSST indicates overall SST warming during this period. Although data coverage is limited in the late 19^th^ century, it is clear that LSATs over regions such as Europe, Russia, India, and the United States exhibit positive trends. If we juxtapose Berkeley Earth land temperature and COBE-SST2 without merging LSATs and SSTs along coastal grids, distinct boundaries between positive and negative trends become evident along the coasts of the US, Europe, and India (Fig. 12e1). In contrast, a pattern more consistent with our expectations would resemble Fig. 12e3, where regions of positive and negative anomalies exhibit continuity across coastlines.

Finally, we examine century-long temperature trends and the evolution of linear trends up to 2023. Long-term trends of global mean surface temperature in DCENT, HadCRUT5, NOAA global temperature, and GISTEMP all increase from around 0.05°C per decade when the starting year is 1850 to approximately 0.2°C per decade when the starting year is 1970 (Fig. 11d). After 1970, the warming rate remains relatively stable, indicating that the ongoing warming since the 1970s follows mostly a linear trend. The spatial pattern of the trend in 1880–2023 is generally consistent across different temperature estimates (Fig. 12a5–e5).

## Usage Notes

DCENT, DCLSAT, and DCSST data are provided in NetCDF format as anomalies relative to the 1982–2014 climatology on a 5° × 5° longitude and latitude grid. This format makes it easy to analyze the data using commonly-used programming languages such as Python, Matlab, and NCL. The DCENT ensemble consists of 200 members, each representing an equally likely realization of the data generation process. As a result, although the ensemble-mean estimate is provided, we encourage users to explore and analyze the entire 200-member ensemble to quantify uncertainty effectively.

The current version of DCENT does not include estimates of sampling and measurement uncertainty or infill for grid cells without direct measurements. Future updates will address these issues. Specifically, forthcoming versions will add estimates of sampling and measurement uncertainties, based on the number of measurements or stations in each grid cell, as highlighted in relevant literature. Additionally, a machine learning-based algorithm for data mapping is under development to introduce spatially infilled temperature products, improving its utility for AMIP-type model simulations. Subsequent updates will focus on providing long-term sub-monthly temperature analyses at a spatial resolution of 1 degree or finer, aiming to support studies on climate extremes and risk management more effectively. These planned improvements demonstrate a continuous effort to improve and expand the DCENT dataset for the benefit of the scientific community.

## Data Availability

The complete method is detailed in the manuscript, and the corresponding code is available at https://github.com/duochanatharvard/DCENT. All packages are developed in Matlab and have been tested on both MacOS and Linux systems, using Matlab version 2021a.
